# Complications, Conversion, and Secondary Procedures Following Minimally Invasive Periacetabular Osteotomy: A Single-Surgeon Case Series

**DOI:** 10.1016/j.artd.2025.101766

**Published:** 2025-07-05

**Authors:** Mohammad H. Amer, Yash Pursun, Christian Smith, Karadi H. Sunil Kumar, Ajay Malviya

**Affiliations:** a Orthopaedic Department, Northumbria Healthcare NHS Foundation Trust, Northumbria House, Cobalt Business Park, Newcastle Upon Tyne, UK; b Cairo University, Al Kasr Al Aini, Cairo Governorate, Egypt; c Newcastle University Medical School The Medical School, Newcastle University, Newcastle upon Tyne, UK; d Guy's and St Thomas' NHS Foundation trust, St Thomas' Hospital, London, UK; e Cambridge University Hospitals NHS Foundation Trust, Addenbrooke's Hospital, Cambridge, UK; f Medical School, Sunderland University, Sunderland, UK

**Keywords:** Periacetabular osteotomy, Complications, Risk factors

## Abstract

**Background:**

The introduction of minimally invasive periacetabular osteotomy (PAO) has reduced complications, allowing a broader range of patients to be considered for the procedure. This study aimed to identify patient-specific risk factors for poor outcomes.

**Methods:**

This retrospective case series (n = 513) used data from a local hip registration registry. Isolated PAOs with at least 1-year follow-up were included. Electronic records were reviewed to extract demographics and variables. The primary outcome was complication rate, with secondary outcomes including secondary procedures and conversion to total hip arthroplasty. Logistic regression was performed to correlate independent variables to outcomes, and Kaplan-Meier analysis assessed the survival of the native hip and cumulative complication risk.

**Results:**

Complication rate was 6.2%. Higher body mass index (BMI), smoking, Tönnis grade 2, and increasing age were associated with higher odds of complications (*P* < .05). The nonunion rate was 4.3%; higher BMI and age linked to increased risk (*P* < .05). 10.9% of patients required a secondary procedure and cumulative risk for secondary procedures at 5 years was 11.4% and at 10 years was 17.2%. BMI correlated with the need for secondary procedures (*P* = .001). 3.7% (n = 19) required conversion to total hip arthroplasty with a mean time to conversion of 4.6 years ±2.04. The 5- and 10-year survival rates were 96.3 and 92.7%, respectively.

**Conclusions:**

Minimally invasive PAO has acceptable rates of complication and conversion at mid-term follow-up. Age, BMI, smoking status, and Tönnis grade 2 are associated with inferior outcomes. Knowledge of patient-specific risk factors can help in decision-making, expectation setting, and perioperative interventions.

## Introduction

Periacetabular osteotomy (PAO) is a well-established technique to correct acetabular dysplasia, whether primary or secondary with good clinical outcomes [[1], [2], [3], [4], [5]]. The intended benefit of PAO is to preserve a well-functioning painless hip, avoiding/delaying a total hip arthroplasty (THA), in a group of patients comprising predominantly young people with nonarthritic hips. A THA provides excellent pain relief but carries the risk of multiple revisions in one’s lifetime and lower clinical effectiveness in young and active individuals [6,7].

The benefit of PAO must be balanced against the risks. Authors have reported a major complication rate of up to 37% [8]; albeit the risk is much lower with minimally invasive (MIS) PAO [9,10]. MIS PAO is a modification of the Smith-Petersen approach, and the steps have been detailed in the methodology. A complication or an unfavorable outcome may necessitate a secondary intervention which adds to the surgical burden and prolongs recovery. This may be in the form of hip arthroscopy, fixation of nonunion, or a conversion to THA [11].

We aim to evaluate the outcome of a large single-surgeon case series of MIS PAOs, with a focus on the following study questions:

1. What are the complications and complication rate for patients undergoing MIS PAO and risk factors associated with it?
2. What are the secondary procedures, the rate, cumulative risk at 5 and 10 years, and risk factors associated?
3. What is the conversion rate to THA, mean time to conversion, overall survival at 5 and 10 years, and risk factors associated?

## Material and methods

### Study design

A retrospective service evaluation of prospectively collected data in the local hip preservation registry between January 2013 and October 2022 was performed. The study received Caldicott approval and was registered with the local audit department. All patients were consented for data collection and access preoperatively.

We focused on identifying cases of isolated PAOs for hip dysplasia with a minimum follow-up of 1 year. PAOs performed concurrently with de-rotational osteotomies and surgical hip dislocations were excluded. Indications for surgery were all patients with symptomatic dysplasia (including borderline dysplasia) or acetabular retroversion, as proven by standard radiographs and computed tomography (CT) scans, who had failed to respond to nonoperative treatment. Exclusion criteria for surgery were all patients with reduced joint space or chondropathy on imaging and patients over the age of 50. Tönnis grade I and II with preserved joint space were included.

Dysplasia was diagnosed based on the contemporary definition proposed with Wilkin et al. [12], while retroversion was diagnosed using clinical features of femoroacetabular impingement and the presence of all 3 radiological signs of retroversion with normal lateral central-edge angle (LCEA) [13].

Patients’ records were reviewed and variables including demographic information, body mass index (BMI), smoking status, and prior ipsilateral open hip surgery and/or hip arthroscopy were documented for analysis.

Patients’ preoperative imaging was reviewed by orthopaedic consultants involved with the project, with assistance from senior radiologists. Input variables included radiological diagnosis, Tönnis grade [14], LCEA [15], acetabular index (AI) [16], acetabular version (AV) [17], and femoral version (FV) [18]. Postoperative radiographs were analyzed to calculate postoperative LCEA, AI, and magnitude of change. Patients graded Tönnis 2 all had acetabular cyst(s) which was/were not communicating with the joint and had no visible reduction of joint space or evidence of chondropathy on the magnetic resonance imaging (MRI) and CT scan.

### Secondary procedures

The decision to offer secondary procedures was based on a failure of PAO for symptomatic control. Secondary procedures included femoral osteotomies, open exploration, refixation, etc. THA was not considered a secondary procedure. Any surgical intervention following PAO was included as a secondary procedure barring routine metalwork removal and injections.

If patients had persistent pain after PAO with a labral tear or residual impingement pathology and no chondral degeneration on MRI arthrogram, they were offered a hip arthroscopy. Patients with reduced joint space and progression of osteoarthritis as per Tönnis grade and chondropathy on MRI arthrogram were offered a THA.

### Description of MIS PAO

**Figure 1:**
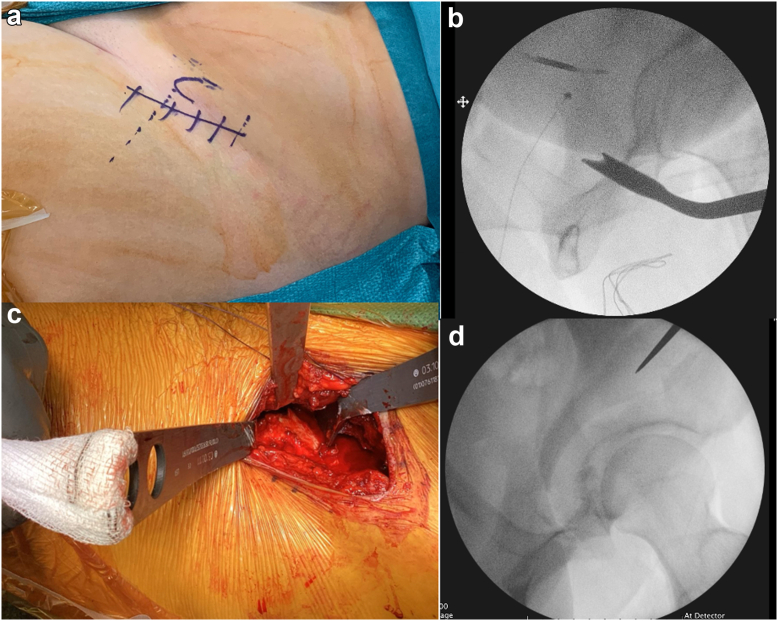
(a-d) (a) 8-10 cm skin incision 1 finger breadth below and parallel to iliac crest, (b) Iliac oblique view using II showing ischial osteotomy performed, (c) Intraoperative photo showing positioning of bi-pronged angle retractor and 2 Hohman’s retractors for pubic osteotomy protecting obturator neurovascular bundle, (d) Iliac oblique view using II showing start point of posterior column cut.

MIS PAO was performed for all patients by the senior surgeon or done under his close supervision using a well-described technique [19]. Under general anesthetic and a spinal, patients were operated on in a supine position on a radiolucent table with ipsilateral arm crossed and held over an L bar at shoulder level. Intravenous teicoplanin and gentamicin is administered 30 minutes prior to skin incision, 2 grams of intravenous tranexamic acid and cell salvage is used to minimize blood loss and need for allogenic blood products. The whole operation is performed under fluoroscopic control. A bikini line incision around 8-10 cm was used one finger breadth below and parallel to the iliac crest starting from a point one finger breadth medial to the anterior superior iliac spine (ASIS) (Fig. 1a). The fascia over the Tensor Fascia Lata (TFL) was identified and incised longitudinally in a distal to proximal direction toward ASIS. The TFL was retracted medially within its sheath and the superficial fascial incision extended proximally to detach the sartorius, inguinal ligament, and external oblique off the ASIS and iliac crest.

A Hohmann’s retractor was placed over the pelvic brim. The plane between the rectus femoris laterally and iliopsoas medially was developed by dividing the bed of TFL fascia distally. The iliopsoas and iliocapsularis were reflected medially, and the infracapsular recess identified and accessed.

A bifid Ganz angled osteotome was introduced into this space and, under image intensifier (II) guidance, the ischial cut was performed (Fig. 1b). The superior pubic cut was then performed after protection of obturator neurovascular bundle with a bi-pronged angle retractor placed over the superior pubic ramus and a retractor on either side of the bone (Fig. 1c). The posterior column (PC) was then performed under II guidance, starting from the midpoint between the greater sciatic notch and acetabulum, down to the ischial cut (Fig. 1d). This was followed by the iliac cut which starts from below ASIS and meets the PC cut.

The acetabulum was then free, allowing movement in all planes. The required correction was achieved according to the underlying pathology and the acetabular fragment was fixed using 3 or 4 4.5 mm cortical screws. Finally, a synthetic bone graft substitute was used to fill osteotomy gap.

After MIS PAO, all patients followed a standard rehabilitation protocol. Patients were allowed toe-touch weight bearing (WB) for 6 weeks, followed by 2 weeks of 25% partial WB, then 2 weeks 50% partial WB, followed by full WB afterward. Low-molecular-weight heparin was prescribed for all patients for 4 weeks postoperatively unless there was a contraindication.

### Outcome measures

The primary outcome measure for this study was the complication rate. A complication was defined as any adverse effect related to surgical procedure and graded according to the modified Clavien-Dindo grading system [20]. Grades III, IV, and V were included. The secondary outcome measures were the secondary procedure rate, rate of conversion to THA, and survival probability at 5 and 10 years.

### Data analysis

Graph Prism 10.0 software (GraphPad Software Inc. and headquartered in San Diego, California, USA) was used to perform data analysis. Descriptive statistics as mean and standard deviation were used for normally distributed data. Logistic regression was performed to detect any correlation, quantified by the correlation coefficient B, between input variables and dependent outcomes. B refers to the unstandardized regression coefficient from the regression model, which differs from the Pearson’s correlation coefficient ‘r’, as it represents the magnitude of change in the outcome variable per unit change in the predictor. A *P* value <.05 was used to determine statistical significance. Variables found to correlate with statistical significance were used in a regression model to predict probability using DataTab software. Survival analysis was performed, and 95% confidence interval was calculated using asymmetrical methods.

## Results

**Figure 2:**
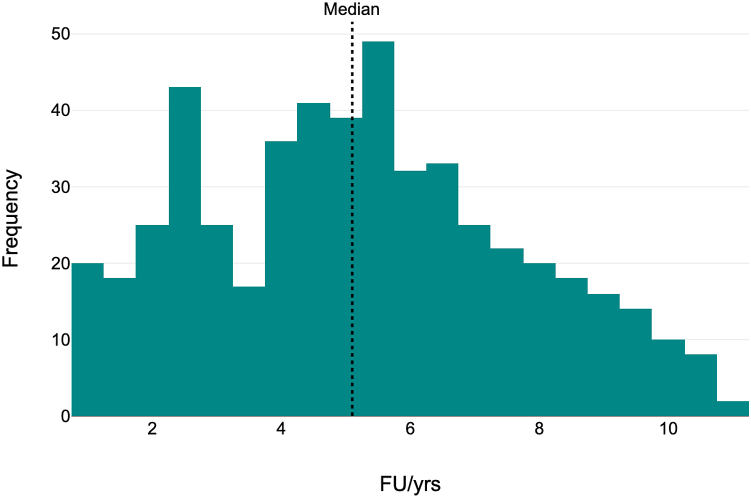
Histogram showing follow-up (FU) distribution in years.

A total of 519 consecutive isolated PAOs were identified during the study period. Six were lost to follow-up, leaving 513 included in the study. Patients were reviewed clinically and radiologically at 6 weeks, 3 months, 1 year, and then annually. The mean follow-up period was 5.18 years ±2.42, median follow-up period 5.1 years, with the minimum follow-up being 1 year (Fig. 2).

The average age was 32.48 years ±9.87. Ninety-three percent (n = 479) were females. The average BMI was 26.54 ± 4.35. Nine percent (n = 47) were smokers. 5.3% (n = 27) had previous open surgery and 13.6% (n = 70) had a prior hip arthroscopy. 92.8% (n = 476) were diagnosed as dysplastic, 5.3% (n = 27) as acetabular retroversion causing femoroacetabular impingement, and 1.9% (n = 10) had PAO for dysplasia due to a childhood hip disorder (such as Perthes disease or postseptic epiphysistis dysplasia). Preoperatively, 12.3% (n = 63) were graded as Tönnis 2 while 40.7% (n = 209) and 47.0% (n = 241) were graded as Tönnis 0 and 1, respectively (Table 1).

**Table 1:** Cohort characteristic.

Feature	Cohort (n = 513)
Age, y	32.48 ± 9.87
Sex, females	93.4%
BMI, kg/m^2^	26.54 ± 4.35
Smoker	9.2%
Diagnosis	
Dysplasia	92.8%
Acetabular retroversion with FAI	5.3%
Congenital dysplasia	1.9%
Tönnis grade	
Grade 0	40.7%
Grade 1	47.0%
Grade 2	12.3%
Previous arthroscopy	13.6%
Previous open hip surgery	5.3%

FAI, femoroacetabular impingement.

**Figure 3:**
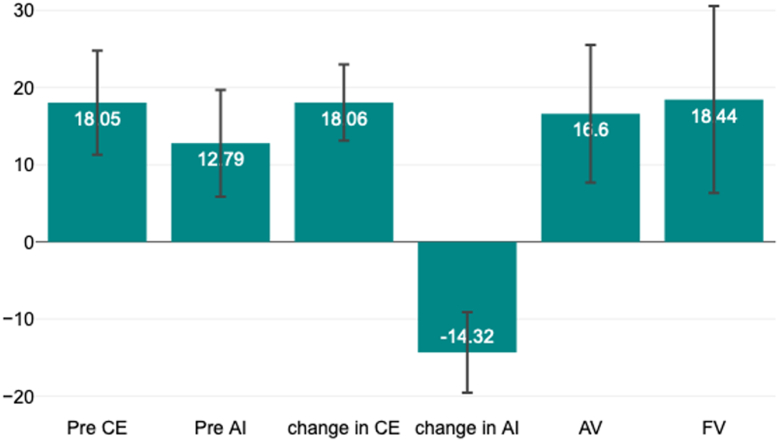
Bar chart showing mean and standard deviation of input radiological variables: preoperative LCEA (pre-CE), preoperative AI (pre-AI), magnitude of change in LCEA (change in CE), magnitude of change in AI (change in AI), AV, and FV.

The preoperative LCEA was 18.05° ± 6.75°, preoperative AI was 12.79° ± 6.92°. The mean AV at femoral head equator was 16.6° ± 8.9°. The mean FV was 18.44° ± 12.12. Postoperatively, the mean LCEA was 36.11° ± 5.04° with a mean change of 18.06° ± 4. 95°. The mean AI improved to −1.53° ± 5.99° SD with a mean change of −14.3° ± 5.23° SD (Fig. 3).

### Complications

6.4% (n = 33) sustained a complication(s) (Table 2). One case of nonfatal pulmonary embolism was categorized as grade IV. No grade V complications were observed. 1.2% (n = 6) suffered a postoperative infection requiring an intervention. No patient suffered a major vascular injury or any injury to the femoral, sciatic, or obturator nerve. Lateral femoral cutaneous nerve injuries were not recorded. Higher BMI (B = 0.17; *P* < .001), smoking (B = 1.3; *P* = .013), Tönnis grade 2 (B = −2.37; *P* = .04), and increasing age (B = 0.1; *P* = .002) were identified as significant risk factors for any complication (Table 3). Smoking increased the odds of having a complication by 3.67 times. A regression model was applied to represent both extremes of the spectrum: the complication risk for a 23-year-old nonsmoker with a BMI of 23 and Tönnis grade 0 would be 1%, compared to 11% for a 40-year-old smoker with a BMI of 35 and Tönnis grade 2.

**Table 2:** Complications following MIS PAO by type, number, and percentage.

Complication	Number	Percentage (%)	Clavien-dindo classification
Nonunion	22	4.3	III
Infection	6	1.2	III
Thromboembolism	3	0.6	III/IV
Failure of fixation	1	0.2	III
ASIS fracture	1	0.2	III

**Table 3:** Logistic regression analysis for risk factors for complications following isolated PAOs.

Feature	Coefficient B	Standard error	z	*P* value	Odds ratio	95% confidence interval
Constant	−12.57	2.95	4.26	<.001	0	0-0
Pre-CE	0	0.07	0.05	.958	1	0.87-1.14
Pre-AI	−0.03	0.06	0.5	.621	0.97	0.87-1.09
Change in CE	0.06	0.06	1.01	.31	1.06	0.94-1.2
Change in AI	−0.07	0.05	1.21	.226	0.94	0.84-1.04
Tonnis 1	−0.17	0.5	0.34	.735	0.85	0.32-2.23
Tonnis 2	−2.37	1.15	2.06	.04	0.09	0.01-0.89
AV	0	0.03	0.18	.855	1	0.96-1.06
FV	0	0.02	0.16	.876	1	0.97-1.04
PAO for retroversion	0.61	1.24	0.49	.625	1.83	0.16-20.72
PAO for other	1.34	1.46	0.92	.36	3.8	0.22-66.39
Age AT OP	0.1	0.03	3.12	.002	1.1	1.04-1.17
Male	−1.35	1.12	1.21	.227	0.26	0.03-2.32
BMI	0.17	0.05	3.73	<.001	1.19	1.08-1.3
Smoking	1.3	0.52	2.48	.013	3.67	1.31-10.23
Prior open surgery	1.23	0.98	1.25	.212	3.42	0.5-23.46
Prior hip arthroscopy	−0.5	0.79	0.63	.528	0.61	0.13-2.88

Pre, preoperative; OP, operation.

Symptomatic delayed/nonunion was observed in 4.3% (n = 22), correlating with higher BMI (B = 0.16; *P* = .001) and increasing age (B = 0.11; *P* = .003). Smoking was not correlated with a higher risk of nonunion (*P* = .842). Ten patients had nonunion of pubic osteotomy and PC stress fracture. 8 patients developed nonunion of pubic osteotomy, ischiopubic (IP) stress fracture, and PC stress fracture. Two patients had nonunion of pubic and IP stress fracture. One patient developed nonunion of iliac osteotomy and 1 patient had nonunion of isolated PC stress fracture.

### Secondary procedures

**Figure 4:**
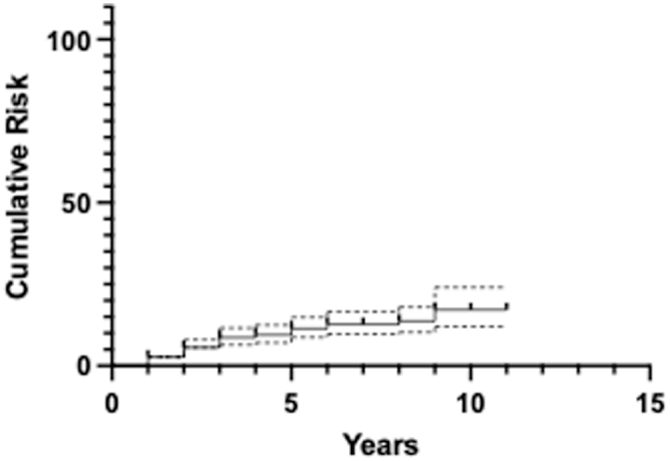
Graph showing cumulative risk for secondary procedures following PAO per year.

10.9% (n = 56) required secondary procedure(s) and 0.97% (n = 5) were on the waiting list (WL) at the time of study completion. Indication for secondary procedures was failure of PAO for symptomatic control and was guided by a combination of clinical, radiographic, and patient-specific factors. Six patients underwent 2 secondary procedures for a total of 62 secondary procedures (Table 4). Higher BMI was the only statistically significant risk factor correlating with the need for secondary surgery (B = 0.11; *P* = .001). A patient with a BMI over 30 had 2.1 times the risk of a patient with BMI <30. The cumulative risk for secondary procedures including conversion to THA at 5 and 10 years were 11.4 (95% CI, 8.8-14.9) and 17.2% (95% CI, 12.1-24.1), respectively (Fig. 4).

**Table 4:** Secondary procedures type, number, and percentage of patients.

Secondary procedure	Number	Percentage including WL (%)
Hip arthroscopy	20 (+1 on WL)	4.1
THA	19	3.7
Nonunion	11 (+3 on WL)	2.7
Debridement for infection	6	1.2
Femoral osteotomy	2 (+1 on WL)	0.6
Open exploration	2	0.4
Refixation for early failure	1	0.2
ASIS fixation	1	0.2

3.9% (n = 20) required a hip arthroscopy postoperatively and 1 patient is on the WL. Of the 20 patients undergoing secondary hip arthroscopy, 4 were converted to THA. 2.14% (n = 11) had secondary procedures for nonunion and 0.6% (n = 3) are still awaiting surgery. Two patients required secondary femoral de-rotational osteotomies and one patient is on the WL.

### Conversion to THA

**Figure 5:**
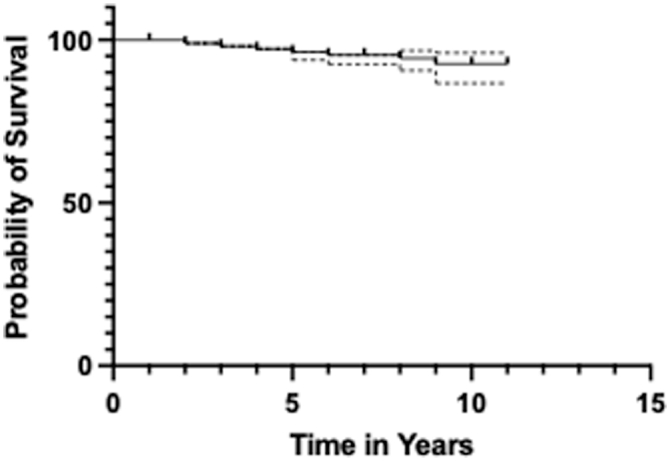
Graph showing survival probability of native hip in years following PAO.

**Figure 6:**
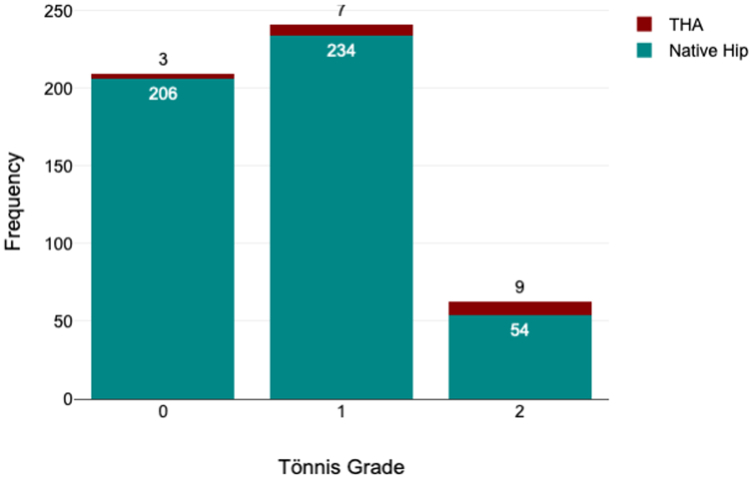
Frequency bar chart showing conversion to THA by Tönnis grade.

3.7% (n = 19) required conversion to THA with a mean time to conversion of 4.6 years ±2.04. The 5- and 10-year survival rates were 96.3 (95% CI, 93.9-97.8) and 92.7% (95% CI, 86.7-96.0), respectively (Fig. 5). Tönnis grade 2 was the only statistically significant risk factor associated with conversion to THA (B = 2.04; *P* = .014) having an odds ratio of 7.89 (Fig. 6).

## Discussion

In this article, we have presented a large case series of consecutive isolated PAOs by a single surgeon. The complication rate, conversion to THA rate, and secondary procedure rate has been reported, filling a gap in the literature. We have identified age, BMI, smoking, and Tönnis grade 2 as risk factors for inferior outcomes. The complication risk varies between 1 and 11% based on these variables.

Our series included dysplasia, retroversion, and secondary dysplasia following childhood disorders. The diagnoses were made preoperatively based on clinical and radiological findings rather than discrete radiological findings which are known to be heterogeneous and not fully reflective of entire spectrum of pathologies [12,13,21]. Dysplasia was presented as a single group, rather than subdividing into more ambiguous categories such as borderline dysplasia [12]. In our series, preoperative diagnosis, LCEA, AI, and AV did not have a statistically significant effect on the complications, secondary procedure rate, or conversion to a THA.

The main strength of this study is the large sample size and medium-term follow-up. The operative technique and postoperative protocol were standardized throughout the study period thus eliminating intervention- and surgeon-related factors as confounders.

### Complications

The overall major complication rate in our cohort (6.4%) was comparable to previous studies by Ali et al. (7%) and Zaltz et al. (5.9%) [9,22]. While the academic network of conservational hip outcomes research group study by Zaltz et al. included 205 patients treated by 10 surgeons across 7 centers, our findings demonstrate similar outcomes in a single-surgeon setting [22]. This study reported on 5 types of grade III and above complications compared to 4 in the academic network of conservational hip outcomes research group study. Grade I and II complications were not included due to retrospective nature of this study; these complications are mostly handled by primary care and in outpatient setting and including them caries a risk under-reporting and information bias.

Increasing age, higher BMI, Tönnis grade 2, and smoking were identified as statistically significant risk factors to have a complication. In the current literature, age > 40 and BMI >30 are associated with an increased risk of complications [[23], [24], [25]]. A regression model was used to predict the complication risk of an ideal patient and a patient with the worst combination of which gives a framework that can be used to create a patient-specific risk.

**Figure 7:**
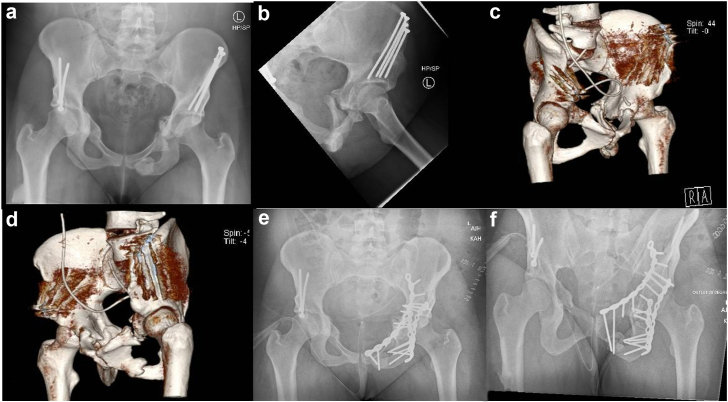
Imaging of a 28-year-old female. (a) Pelvis AP showing nonunion of pubic osteotomy and IP stress fracture, (b) Iliac oblique showing nonunion of PC stress fracture, (c) CT 3D reconstruction showing PC and pubic nonunion, (d) CT 3D reconstructions showing pubic and IP nonunion, (e) Pelvis AP showing postfixation construct performed through anterior intrapelvic approach and Kockher-Langenbeck, (f) Pelvis outlet view showing screws crossing IP stress fracture. 3D, 3-dimensional; AP, anterior-posterior.

The most common complication observed was symptomatic nonunion at 4.3%. The nonunion occurs at an osteotomy site and/or of a stress fracture [26]. In most patients, nonunion occurs at 2 or 3 sites creating a free IP or public fragment that moves independently under control of the hamstring and adductor muscles, interfering with stability needed for union (Fig. 7). Nonunion was correlated with an increased age and higher BMI. Matsunaga et al. identified smoking as a risk factor for delayed union of pubic osteotomy with an odds ratio of 10.7 [27]. Previous studies reported a nonunion rate between 2.2 and 8% [9,28].

Selberg et al. noted that there were no difference in patient-reported outcome measures (PROMs) between patients with union and those with nonunion [28]. This explains the discrepancy between nonunion and secondary procedures for nonunion in our series as not all patients were symptomatic enough to undergo fixation. Selberg et al. also identified increasing age and BMI as risk factors for nonunion with the addition of severity of dysplasia which was not replicated in our series [28].

### Secondary procedures

The secondary procedure rate was 10.9% with the most common being hip arthroscopy. BMI was the only factor to correlate with secondary procedures. 3.9% (n = 20) had residual groin pain, positive flexion-adduction-internal rotation sign, imaging suggestive of a cam deformity deformity or a labral tear, or a positive response to injection. These patients were offered secondary hip arthroscopy. Laboudie et al. reported a secondary hip arthroscopy rate of 7.4%. Like our series, they reported a high rate of conversion to THA following secondary hip arthroscopy, with no improvement in PROMs [29]. A femoral de-rotational osteotomy was required in 2 patients and one patient is on the WL. Femoral version was not associated with increased complications, secondary procedures, or conversion. Goronzy et al. reported that FV did not affect PROMs [30]. It is the senior author’s preference to correct acetabular coverage first in the presence of increased FV. A femoral de-rotational osteotomy was performed in the same sitting of the PAO in the presence of locked external rotation in the prone position.

### Conversion to THA

**Figure 8:**
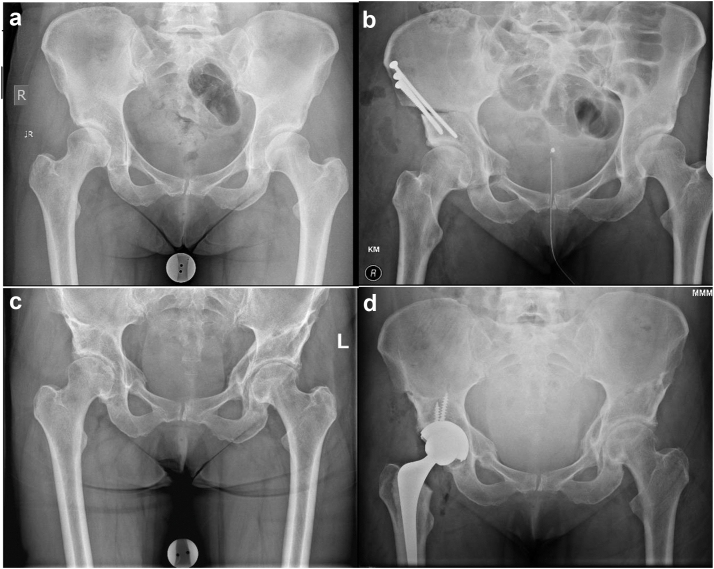
Plain pelvis and both hips AP x-rays. (a) Preoperative x-rays at age of 46 years, (b) Immediate postoperative right PAO, (c) 8 years post right PAO, (d) Right THA postoperative x-rays at 9 years post-PAO. AP, anterior-posterior.

The overall conversion rate to THA was 3.7% (Fig. 8). The medium-term overall survival compares similarly to previous publications [4,11]. Our series included Tönnis grade 2 hips with acetabular cysts which did not communicate with the joint. These were included only after advanced imaging in the form of CT scan and MRI scan had ruled out chondropathy and reduction of joint space. We believe that in anteriorly located acetabular cysts, following PAO correction the cysts would potentially move outside of WB zone acting as an offloading osteotomy like high tibial osteotomies. Zhang et al. reported that there was no difference between Tönnis grade 1 and 2 in terms of functional scores [31]. However, performing PAOs in patients with Tönnis grade 2 changes carries an increased risk of conversion to a THA, a finding reported by Millis et al., and replicated in this study [32]. This may be slightly confounded by a lower threshold to offer a THA for Tönnis 2 hips compared to grade 1 and 0.

### Limitations

PROMs were not presented even though they are routinely collected. Low compliance in returning postoperative PROMs creates an attrition bias and hence the study was limited to binary outcomes. Factors known to correlate with complication rate such as vitamin D deficiency were not included in logistic regression analysis as this was not routinely assessed preoperatively/postoperatively [33]. As with all retrospective case series, there is a potential for selection bias in terms of patient selection for surgery. Under-representation of older patients, males, and smokers is inevitable as studied pathologies affect predominantly females and smoking was a relative contraindication to osteotomy procedures because of risk of nonunion [34,35]. Finally, it should be stated that, as this is a single-surgeon case series, the results may not be fully generalizable to other surgeons or institutions.

## Conclusions

MIS PAO has demonstrated an acceptable rate of complications and conversions to THA at mid-term follow-up, even with extended indications. The study highlights that age, BMI, smoking status, and Tönnis grade 2 are significant risk factors associated with poorer clinical outcomes, including higher complication rates, increased need for secondary procedures, and a higher likelihood of conversion to THA. Specifically, older age, elevated BMI, smoking, and advanced Tönnis grade were all linked to an increased risk of complications such as symptomatic nonunion and delayed recovery, as well as the need for additional surgical interventions.

This knowledge of patient-specific risk factors is helpful for refining preoperative assessment and counseling, as it allows for more individualized decision-making. Surgeons can use these insights to optimize clinical outcomes, set realistic expectations, guide discussions about potential risks with patients, and implement neoadjuvant strategies to mitigate modifiable risk factors. Future studies with a focus on preoperative interventions, PROMs, and long-term follow-up will further refine the role of MIS PAO in the management of hip dysplasia and related pathologies.

## Ethical statement

This was a service evaluation project with no ethical committee approval required, registered locally at Northumbria Healthcare NHS trust with reference number 8664.

## Conflicts of interest

A. Malviya has Medical/Orthopaedic publications editorial/governing board at Journal of Hip Preservation Surgery–Deputy Editor and Editorial Board member–AJSM, VJSM; and is a board member/committee appointments for British Hip Society–Treasurer and British Orthopaedic Association Ed Car committee–Vice Chair. K.H. Sunil Kumar is a member for BHS Education Committee and Lead Editor for Trauma–UKITE; all other authors declare no potential conflicts of interest.

For full disclosure statements refer to https://doi.org/10.1016/j.artd.2025.101766.

## CRediT authorship contribution statement

**Mohammad H. Amer:** Writing – original draft, Software, Formal analysis. **Yash Pursun:** Writing – review & editing, Visualization. **Christian Smith:** Methodology, Data curation. **Karadi H. Sunil Kumar:** Supervision, Conceptualization. **Ajay Malviya:** Validation, Supervision, Resources, Project administration, Investigation, Conceptualization.

## Appendix A Supplementary data

Conflict of Interest Statement for Malviya

Conflict of Interest Statement for Smith

Conflict of Interest Statement for Sunil Kumar

Conflict of Interest Statement for Amer

Conflict of Interest Statement for Pursun
