# Supplementary material for: Complications, Conversion, and Secondary Procedures Following Minimally Invasive Periacetabular Osteotomy: A Single-Surgeon Case Series
Source: Arthroplast Today. 2025 Jul 5;34:101766. doi: 10.1016/j.artd.2025.101766 (PMC12272465; doi:10.1016/j.artd.2025.101766)
Supplement: Conflict of Interest Statement for Malviya [file mmc1.pdf]

INDIVIDUAL CONFLICT OF INTEREST STATEMENT

American Association of Hip and Knee Surgeons

(Adopted from the American Academy of Orthopaedic Surgeons disclosure statement)

The following form **must be filled out completely and submitted by each author (example, 6 authors, 6 forms).**
All items require a response. If there is no relevant disclosure for a given item, enter "*None*."

Complications, conversion, and secondary procedures following minimally invasive periacetabular osteotomy: a single surgeon case series.

1. Royalties from a company or supplier (The following conflicts were disclosed)

None

2. Speakers bureau/paid presentations for a company or supplier (The following conflicts were disclosed)

None

3A. Paid employee for a company or supplier (The following conflicts were disclosed)

None

3B. Paid consultant for a company or supplier (The following conflicts were disclosed)

None

3C. Unpaid consultants for a company or supplier (The following conflicts were disclosed)

None

4. Stock or stock options in a company or supplier (The following conflicts were disclosed)

None

5. Research support from a company or supplier as a Principal Investigator (The following conflicts were disclosed)

None

6. Other financial or material support from a company or supplier (The following conflicts were disclosed)

None

7. Royalties, financial or material support from publishers (The following conflicts were disclosed)

None

8. Medical/Orthopaedic publications editorial/governing board (The following conflicts were disclosed)

Journal of Hip Preservation Surgery – Deputy Editor; Editorial Board member – AJSM, VJSM

9. Board member/committee appointments for a society (The following conflicts were disclosed)

Treasurer – British Hip Society; Vice Chair – British Orthopaedic Association Ed Car committee

Each author must sign AND print or type his/her name, date and submit a separate form
In addition, one BLINDED Conflict of Interest form (no author names used) should be submitted per manuscript with all author disclosures.

Ajay Malviya

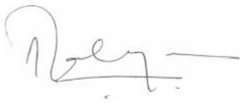

18/11/2024

Author Name (Print or Type) Author Signature Date
